# Gut dysbiosis and systemic inflammation in elderly hypertensive patients with amnestic mild cognitive impairment

**DOI:** 10.3389/fimmu.2026.1871446

**Published:** 2026-06-11

**Authors:** Zongxin Ling, Yiwen Cheng, Xiaocui Xu, Shengyu Huang, Xia Liu, Yangtian Chen, Pingping Hu, Lingbin Wu, Longyou Zhao, Yihua Huang

**Affiliations:** 1 State Key Laboratory for Diagnosis and Treatment of Infectious Diseases, National Clinical Research Center for Infectious Diseases, China-Singapore Belt and Road Joint Laboratory on Infection Research and Drug Development, National Medical Center for Infectious Diseases, Collaborative Innovation Center for Diagnosis and Treatment of Infectious Diseases, The First Affiliated Hospital, Zhejiang University School of Medicine, Hangzhou, Zhejiang, China; 2 Department of Anesthesiology, Affiliated Hospital of Nantong University, Nantong, Jiangsu, China; Medical School of Nantong University, Jiangsu, China; 3 Department of Laboratory Medicine, Wenzhou Medical University, Wenzhou, Zhejiang, China; 4 Department of Intensive Care Unit, The First Affiliated Hospital, Zhejiang University School of Medicine, Hangzhou, Zhejiang, China; 5 Department of Laboratory Medicine, Lishui Second People’s Hospital, Lishui, Zhejiang, China; 6 Department of Nursing, Lishui Second People’s Hospital, Lishui, Zhejiang, China

**Keywords:** dysbiosis, gut microbiota, hypertension, inflammation, mild cognitive impairment, short-chain fatty acids

## Abstract

**Introduction:**

Gut microbial dysbiosis has been linked to both high blood pressure and neurodegeneration, but its involvement in hypertensive patients with amnestic mild cognitive impairment (aMCI) has not been well characterized in this specific population.

**Methods:**

In this cross-sectional investigation, we enrolled 205 older Chinese adults: 52 healthy controls, 83 hypertensive individuals with normal cognition (HTN-CN), and 70 hypertensive subjects with aMCI (HTN-aMCI). Gut microbiota composition was profiled by 16S rRNA sequencing, and serum levels of 27 inflammatory mediators were quantified by multiplex immunoassay.

**Results:**

Compared to the HTN-CN and control groups, the HTN-aMCI group showed not only a greater richness of gut microbes but also a markedly segregated microbial community structure. The HTN-aMCI microbiota was characterized by significant depletion of short-chain fatty acid (SCFA)-producing genera (*Roseburia, Blautia, Faecalibacterium*) and enrichment of opportunistic pathogens (*Streptococcus*, *Clostridium*_*sensu*_*stricto*_1, *Enterococcus*). Co-occurrence network analysis revealed disrupted microbial interactions in HTN-aMCI, and functional prediction showed enhanced lipopolysaccharide biosynthesis and reduced SCFA metabolism. HTN-aMCI patients had elevated pro-inflammatory cytokines (IL-1β, IL-6, IL-8, IL-17, IP-10, RANTES). Notably, after FDR correction, *Blautia* abundance correlated negatively with inflammatory markers and positively with cognitive scores, whereas pathobionts showed opposite patterns (all q < 0.05).

**Discussion:**

These findings indicate that hypertensive individuals with aMCI harbor a specific gut microbial dysbiosis marked by loss of SCFA producers, expansion of pathobionts, and disrupted microbial networks, which together associate with systemic inflammation and cognitive decline. Our results support the notion that targeting gut microbiota might represent a potential therapeutic avenue for hypertension-related cognitive impairment.

## Graphical Abstract

**Figure:**
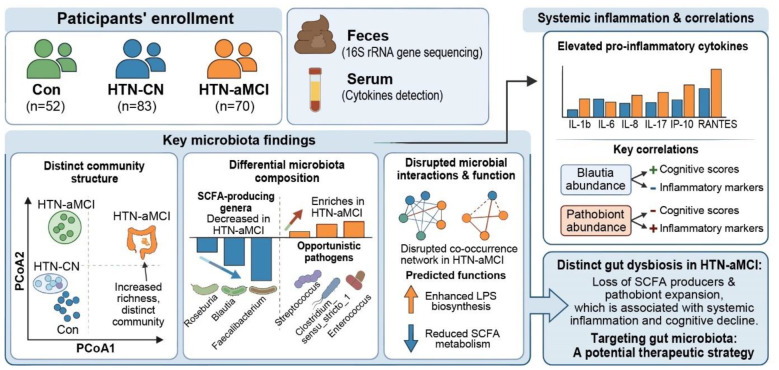

## Introduction

Amnestic mild cognitive impairment (aMCI) involves a memory decline exceeding age-related expectations without major interference with daily activities, positioning it as a key precursor to Alzheimer’s disease (AD) (1). As the global population ages, the prevalence of aMCI among older adults has risen substantially, creating a pressing public health challenge and underscoring the need to identify modifiable risk factors and underlying biological mechanisms for early prevention and intervention (2).

Hypertension (HTN) is highly common among the elderly, especially in China, and is well recognized as an independent risk factor for cognitive deterioration and dementia, including aMCI and AD (3, 4). The mechanisms linking HTN to cognitive impairment involve cerebrovascular damage (e.g., microangiopathy), chronic systemic inflammation, and oxidative stress, which collectively impair cerebral blood flow and neuronal function (5). However, the full spectrum of its influence, particularly in combination with other biological systems, remains under investigation. Emerging evidence supports potential bi-directional interaction between HTN and gut microbiota, suggesting that dysbiosis may worsen HTN while elevated blood pressure further modifies microbial composition (6–9). This bi-directional relationship implies that gut microbiota could be associated with the link between HTN and cognitive dysfunction, a hypothesis requiring further exploration.

The gut microbiota, a complex community of microorganisms in the gastrointestinal tract, exerts broad regulatory influences on host physiology, including brain function, via the gut-brain axis (1, 10). Microbial metabolites such as short-chain fatty acids (SCFAs), lipopolysaccharides (LPS), and neuroactive compounds can modulate neuroinflammation, blood-brain barrier integrity, and central nervous system function, thereby affecting cognitive processes (11). Dysbiosis, an imbalance in the gut microbial community, has been implicated in various neurological disorders, including AD and Parkinson’s disease (PD) (12–17). Recent work has specifically shown that older adults with MCI exhibit altered gut microbiota profiles relative to cognitively healthy individuals (18–22). These changes often include altered microbial diversity and shifts in specific bacterial taxa, which may contribute to systemic inflammation and impaired cognition.

Despite growing awareness of links among gut microbiota, HTN, and cognition, the specific microbial alterations in elderly hypertensive patients with aMCI remain poorly defined. China has one of the world’s largest aging populations, with a high prevalence of both hypertension (HTN) and amnestic mild cognitive impairment (aMCI) among the elderly. According to recent national surveys, the prevalence of HTN in Chinese adults aged ≥ 60 years is approximately 55-60% (23), while the prevalence of aMCI in this age group is estimated at 15-20% (24). These high prevalence rates make this population an important research focus. Given that both HTN and gut dysbiosis have each been associated with cognitive decline (5, 25–28), their potential synergistic effects on aMCI pathogenesis are likely significant but remain uncharacterized. To address this gap, the present study systematically characterizes changes in gut microbiota composition and structure in Chinese elderly hypertensive patients with aMCI, comparing them to hypertensive elderly without aMCI and cognitively healthy elderly controls. This investigation aims to provide new insights into the biological mechanisms underlying cognitive decline in this vulnerable population, potentially identifying gut microbial biomarkers and therapeutic targets for aMCI in the context of HTN.

## Methods

### Study population and grouping

As described in our previous work (6), this cross-sectional study enrolled 205 elderly participants (aged ≥60 years) who were community-dwelling and recruited through community health screenings and outpatient clinics in Lishui, China, between May and August 2024. All participants were living independently in the community; none were hospitalized at the time of enrollment. All participants provided written informed consent, and the study protocol was approved by the Ethics Committee of Lishui Second People’s Hospital (approval no. 20230119−01). The cohort included 52 cognitively healthy controls (Con), 83 hypertensive individuals with normal cognition (HTN-CN), and 70 hypertensive individuals with amnestic mild cognitive impairment (HTN-aMCI). HTN was defined according to standard clinical criteria as a systolic blood pressure (SBP) ≥ 140 mmHg and/or a diastolic blood pressure (DBP) ≥ 90 mmHg, or current use of antihypertensive medication, which is consistent with the 2024 Chinese Hypertension Prevention and Treatment Guidelines and the 2024 ESC Guidelines, both of which maintain this definition (29, 30). Detailed information on all antihypertensive medication classes (including CCBs, β-blockers, diuretics, and combinations) was recorded, and no significant differences in any class were observed between the HTN-CN and HTN-aMCI groups. The diagnosis of aMCI was established according to the Petersen criteria and clinical diagnostic guidelines, with a Montreal Cognitive Assessment (MoCA) cutoff score of < 26 indicating cognitive impairment. Cognitive function was assessed using the MoCA to ensure consistent evaluation across groups. Detailed demographic characteristics, medical histories, and medication usage were recorded for each participant. Individuals with major neurological, psychiatric, or gastrointestinal diseases that could potentially confound cognitive or microbiota outcomes were excluded. In addition, participants who had taken antibiotics, probiotics, prebiotics, or any other known microbiota-modulating agents within one month prior to enrollment were excluded. All participants were instructed to maintain their usual dietary habits (all mixed diet) throughout the study period, and no dietary interventions were applied.

### Sample collection

Approximately 2 g of fresh fecal samples were collected using sterile plastic containers and placed into a -80 °C freezer within 15 minutes of collection for subsequent microbiome analysis. Fasting blood samples were drawn in the early morning. After centrifugation at 1500×g for 10 minutes, serum was separated and divided into three sterile 200 µL cryovials, which were stored at −80 °C until cytokine analysis.

### Gut microbiota profiling

Bacterial genomic DNA was extracted from 300 mg of homogenized feces using the QIAamp DNA Stool Mini Kit (Qiagen, Hilden, Germany, Cat. No. 51604), with additional glass−bead beating using a Mini−beadbeater (FastPrep; Thermo Electron Corporation, Boston, MA, USA). The V3−V4 hypervariable regions of the 16S rRNA gene were amplified following established protocols (6, 12, 31–34). Negative controls (lysis buffer and kit reagents only) were included to assess contamination. Library preparation was performed at Hangzhou KaiTai Bio−lab, and sequencing was carried out on the NovaSeq™ 6000 platform (Illumina).

Raw sequencing data were processed with QIIME2 (version 2022.2). After quality filtering, denoising, and chimera removal, amplicon sequence variants (ASVs) were generated using the DADA2 pipeline (35). To account for uneven sequencing depth, the ASV table was rarefied to the minimum library size across all samples prior to downstream analysis. Taxonomy was assigned using the SILVA database (version 138). Alpha and beta diversity metrics were calculated. Linear discriminant analysis effect size (LEfSe) was applied to identify differentially abundant taxa between groups, using the Kruskal−Wallis test followed by pairwise Wilcoxon tests (p < 0.05, LDA score > 3.0) (36). Functional potential was predicted using PICRUSt2 based on the KEGG Orthology database (37, 38).

### Microbial co-occurrence network analysis

To explore ecological interactions among gut bacterial taxa, we constructed co-occurrence networks separately for the Con, HTN-CN, and HTN-aMCI groups. Only genera with a relative abundance > 0.1% in at least 10% of samples within each group were retained to reduce sparsity and avoid spurious correlations. Spearman’s rank correlation coefficients were calculated between all pairs of retained genera. Only statistically significant correlations (p < 0.05, after correction) with an absolute correlation coefficient ρ > 0.3 were considered for network visualization. Networks were visualized using the igraph package in R, where nodes represent bacterial genera and edges indicate significant correlations (red for positive, blue for negative). Node size was scaled to the degree of connectivity (number of significant correlations per genus).

### Cytokine and chemokine measurement

Serum levels of 27 cytokines, chemokines, and growth factors were measured using a Bio-Rad 27-plex multiplex immunoassay (#M500KCAF0Y, Bio-Rad Laboratories, Hercules, CA, USA) according to the manufacturer’s protocol (12, 31–34, 39). The analytes included 16 cytokines, 6 chemokines, and 5 growth factors. Data were collected on a Bio−Plex 200 system and analyzed with Bio−Plex Manager software.

### Statistical analysis

Continuous variables with normal distribution (assessed by Shapiro-Wilk test) and homogeneity of variance (Levene’s test) were compared using one-way ANOVA followed by Tukey’s post-hoc test for pairwise comparisons. Non-normally distributed continuous variables were compared using the Kruskal-Wallis test followed by Dunn’s post-hoc test with Bonferroni correction. Categorical variables (e.g., sex, diabetes, hyperlipidemia, ACEI/ARB use) were compared using the chi-square test. Correlations between microbial genera and cytokines or MoCA scores were evaluated using Spearman’s rank correlation due to the non-normal distribution of microbiome and cytokine data. Statistical significance was set at p < 0.05, with false discovery rate (FDR) correction (Benjamini-Hochberg method) applied for multiple comparisons in differential abundance analysis (LEfSe) and functional pathway comparisons. Analyses were performed using SPSS v24.0 (SPSS Inc., Chicago, IL) and STAMP v2.1.3, with graphics generated using R packages and GraphPad Prism v6.0. Enterotype analysis was performed on genus-level relative abundances using partitioning around medoids (PAM) clustering with Jensen-Shannon divergence. The optimal number of clusters (k = 2-6) was selected based on the Calinski-Harabasz index and silhouette coefficient, and clustering stability was validated by bootstrap resampling (n = 1,000). For PICRUSt2-derived functional pathway comparisons, Welch’s *t*-test was used, and p-values were adjusted for multiple comparisons using the Benjamini-Hochberg FDR method. An FDR-adjusted q-value < 0.05 was considered statistically significant. For the random forest classification, model performance was evaluated using ten-fold cross-validation, which was repeated five times to obtain robust estimates. The cross-validation was stratified by group to preserve the original class distribution in each fold. Variable importance was assessed using the mean decrease Gini index, and the optimal number of features was determined by recursive feature elimination with cross-validation. Receiver operating characteristic (ROC) curves and area under the curve (AUC) analysis (via OECloud tools, https://cloud.oebiotech.com) assessed model performance. All two−sided tests used the Benjamini−Hochberg method for FDR control, with FDR < 0.05 considered significant.

### Accession number

The sequence data from this study are deposited in the GenBank Sequence Read Archive with the accession number PRJNA1261238(https://www.ncbi.nlm.nih.gov/bioproject/PRJNA1261238).

## Results

### Participant characteristics

The clinical characteristics of the three groups are summarized in **Table 1**. Age, sex distribution, and BMI were comparable across groups, indicating that the groups were well balanced for these baseline characteristics. As expected, SBP and DBP were significantly higher in both hypertensive groups (HTN-CN and HTN-aMCI) compared with the Con group (p < 0.001), with no significant difference between the two hypertensive groups. MoCA scores were significantly lower in the HTN-aMCI group than in both the HTN-CN and Con groups (p < 0.001), confirming the cognitive status classification. No significant differences were observed among groups in the prevalence of diabetes or hyperlipidemia (p > 0.05). Among hypertensive participants, the proportion of ACEI/ARB use was similar between the HTN-CN (62.7%) and HTN-aMCI (64.3%) groups (p = 0.837), suggesting that antihypertensive medication use is unlikely to confound the comparisons between these two groups.

**Table 1:** Clinical characteristics of study participants.

Characteristic	Con (n=52)	HTN-CN (n=83)	HTN-aMCI (n=70)	P-value
Age (years)	69.1 ± 4.8	68.7 ± 7.0	70.2 ± 6.8	0.312
Male, n (%)	38 (73.1%)	62 (74.6%)	50 (71.4%)	0.911
BMI (kg/m²)	24.5 ± 1.9	24.1 ± 3.0	24.3 ± 3.1	0.722
SBP (mmHg)	118.5 ± 10.2	138.6 ± 12.4*	140.2 ± 13.1*	<0.001
DBP (mmHg)	74.3 ± 8.1	83.5 ± 9.2*	84.1 ± 9.5*	<0.001
Diabetes, n (%)	6 (11.5%)	18 (21.7%)	16 (22.9%)	0.231
Hyperlipidemia, n (%)	10 (19.2%)	24 (28.9%)	21 (30.0%)	0.342
Mixed diet, n (%)	52 (100%)	83 (100%)	70 (100%)	1.000
Smoking (yes)	12 (23.1%)	22 (26.5%)	18 (25.7%)	0.892
Alcohol (yes)	10 (19.2%)	18 (21.7%)	15 (21.4%)	0.938
ACEI/ARB use, n (%)	–	52 (62.7%)	45 (64.3%)	0.837
MoCA score	27.2 ± 1.5	26.8 ± 1.6	22.4 ± 2.3†	<0.001

Data are presented as mean ± SD for normally distributed continuous variables (Shapiro-Wilk test, p > 0.05 for each group) or as n (%) for categorical variables. SBP, systolic blood pressure; DBP, diastolic blood pressure; MoCA, Montreal Cognitive Assessment; ACEI, angiotensin-converting enzyme inhibitor; ARB, angiotensin receptor blocker. p value for overall comparison across the three groups (one way ANOVA for continuous variables; chi square test for categorical variables). *p < 0.05 vs. Con; †p < 0.05 vs. HTN-CN.

### Altered gut microbiota structure and composition in HTN-aMCI patients

**Figure 1:**
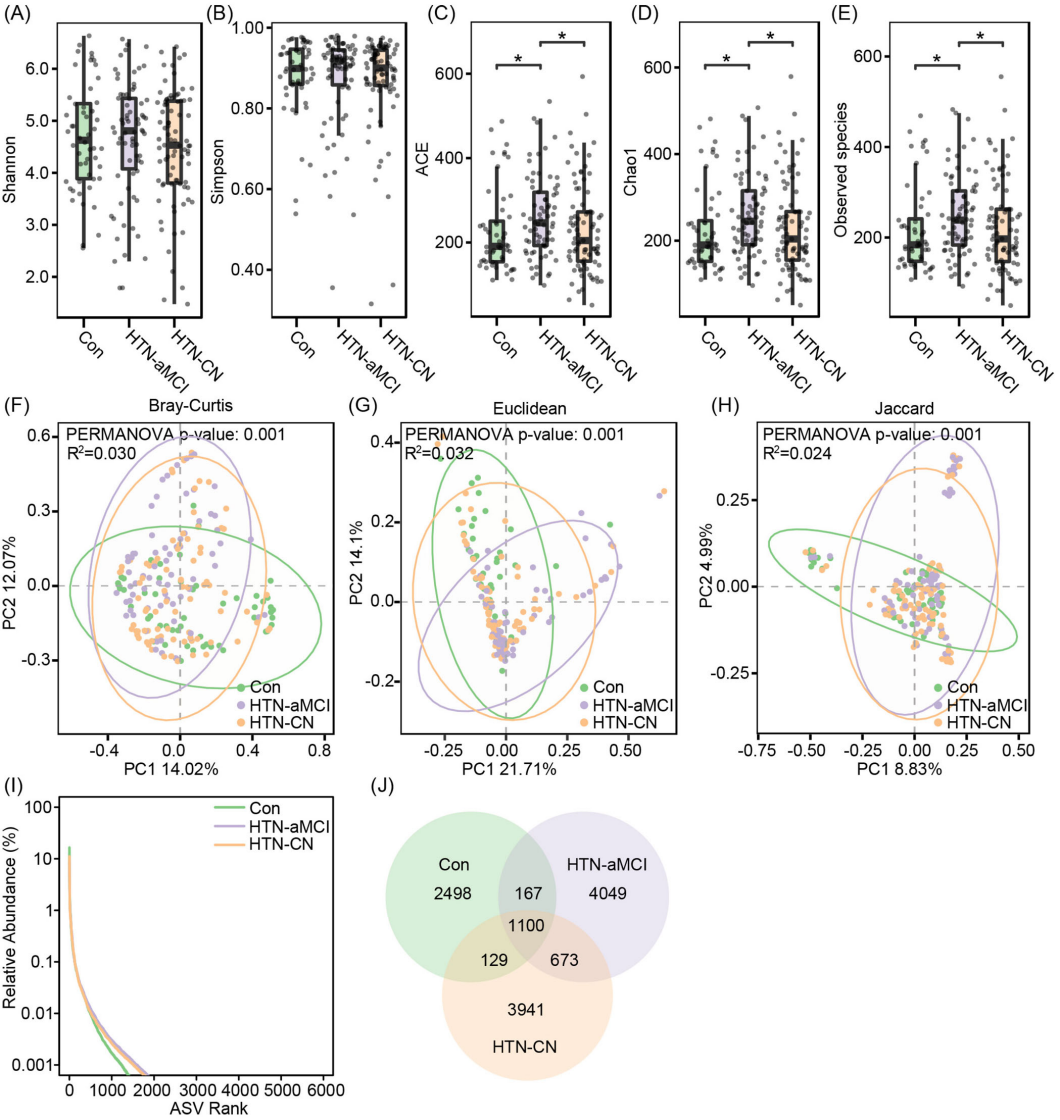
Altered gut microbial diversity and community structure in elderly hypertensive patients with amnestic mild cognitive impairment (aMCI). **(A–E)** Alpha diversity indices comparing the control (Con), hypertensive with normal cognition (HTN-CN), and hypertensive with aMCI (HTN-aMCI) groups. No significant differences were observed in the Shannon **(A)** or Simpson **(B)** indices across groups. However, richness estimators, including ACE **(C)**, Chao1 **(D)**, and observed species **(E)**, were significantly higher in the HTN-aMCI group compared with both the Con and HTN-CN groups (*p < 0.05). Data are presented as mean ± SD. **(F–H)** Principal coordinate analysis (PCoA) plots based on Bray–Curtis **(F)**, Euclidean **(G)**, and Jaccard **(H)** distances, showing clear separation of microbial community structures among the three groups (PERMANOVA, p = 0.001 for all). Each symbol represents an individual sample. **(I)** Rank abundance curves illustrating the distribution of amplicon sequence variants (ASVs) across groups. The Con group exhibited a steeper decline, whereas both hypertensive groups showed more gradual curves, indicating greater species evenness and a higher number of low-abundance ASVs. **(J)** Venn diagram showing the number of unique and shared ASVs among the three groups. The HTN-aMCI group harbored the highest number of unique ASVs, while the Con group had the lowest.

We first assessed gut microbial diversity across the three groups. The overall α-diversity of gut microbiota, as reflected by the Shannon and Simpson indices, did not differ significantly among the three groups (**Figures 1A–B**), indicating comparable community evenness. However, the richness estimators, including ACE, Chao1, and Observed species, were markedly higher in the HTN-aMCI group than in both the control and HTN-CN groups (**Figures 1C–E**, p < 0.05), while no significant difference was observed between Con and HTN-CN groups. These findings indicate that HTN−aMCI exhibited increased species richness without alterations in overall diversity, suggesting an expansion of low−abundance taxa that may reflect ecological disturbance rather than a healthy, stable microbiota. β-diversity analysis using three distance metrics, such as Bray-Curtis, Euclidean, and Jaccard, demonstrated clear separations in microbial community structure among the three groups (PERMANOVA p = 0.001 for all; Bray-Curtis R² = 0.030, Euclidean R² = 0.032, Jaccard R² = 0.024; **Figures 1F-H**), implying distinct microbial profiles particularly associated with the HTN-aMCI group. Rank abundance curves revealed that the Con group exhibited a steeper decline in species abundance, indicating fewer unique ASVs compared with the hypertensive groups. Both HTN-CN and HTN-aMCI groups showed a more gradual decline, suggesting greater species evenness and a higher number of low-abundance ASVs (**Figure 1I**). Venn diagram analysis further demonstrated that the Con group had the lowest number of unique ASVs, whereas the HTN-aMCI group harbored the highest number of unique ASVs, consistent with the rank abundance findings (**Figure 1J**). These findings suggest that HTN is associated with altered gut microbial community composition, and that cognitive decline further drives distinct microbial shifts.

**Figure 2:**
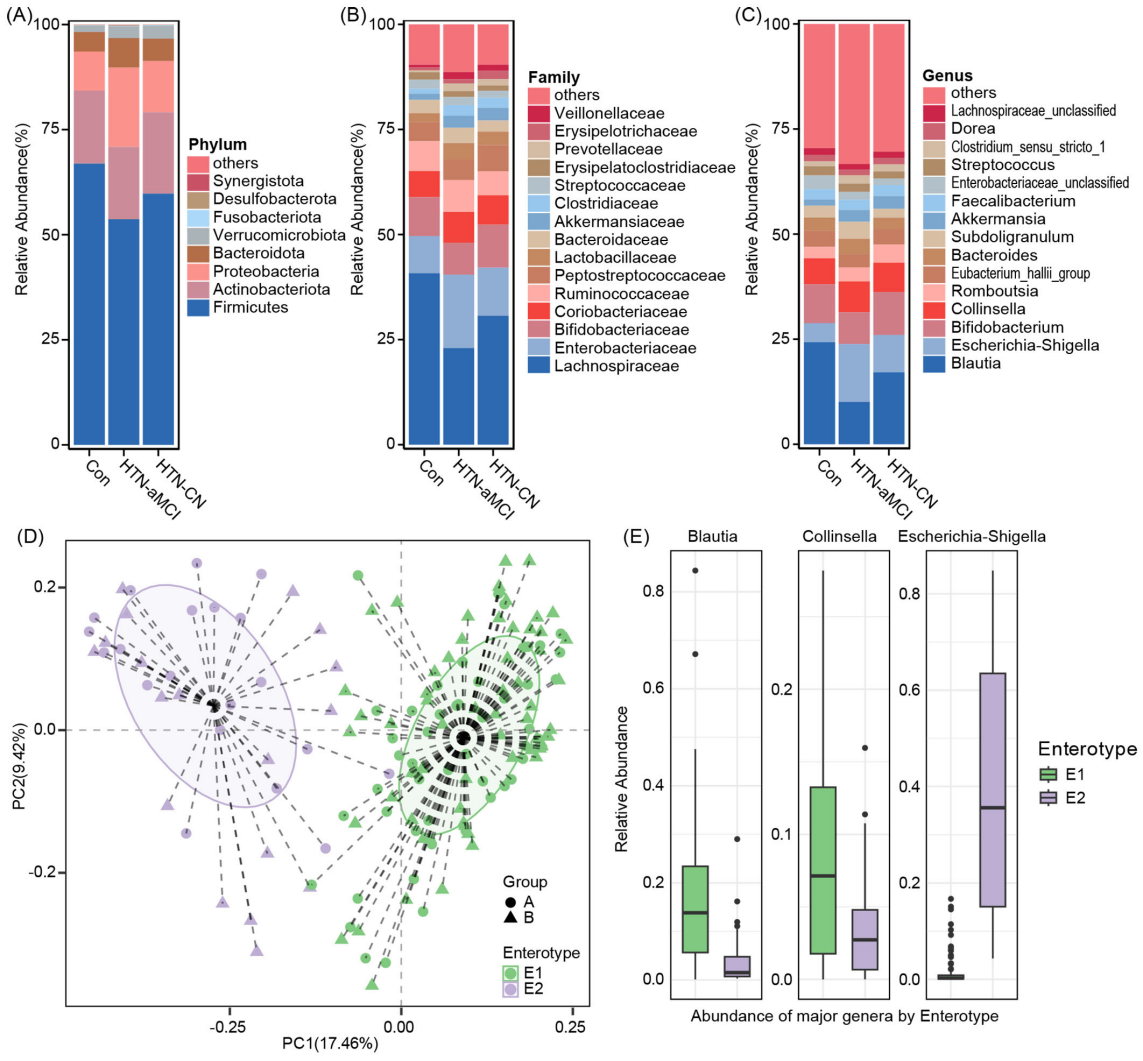
Taxonomic composition and enterotype analysis of gut microbiota in elderly hypertensive patients with amnestic mild cognitive impairment (aMCI). **(A–C)** Bar plots showing the relative abundance of gut microbial communities at the phylum **(A)**, family **(B)**, and genus **(C)** levels across the control (Con), hypertensive with normal cognition (HTN-CN), and hypertensive with aMCI (HTN-aMCI) groups. **(D)** Principal coordinate analysis (PCoA) plot based on genus-level relative abundances, revealing two distinct enterotypes (E1 and E2) among hypertensive participants (HTN-CN and HTN-aMCI combined). Each symbol represents a sample. **(E)** Relative abundance of predominant bacterial taxa within each enterotype.

Consistent with the structural differences, the taxonomic composition at the phylum, family, and genus levels also diverged among groups (**Figures 2A-C**). At the phylum level, Firmicutes Actinobacteriota, Proteobacteria, and Bacteroidota dominated across all groups. At the family level, Lachnospiraceae, Enterobacteriaceae, Ruminococcaceae, and Bacteroidaceae were the predominant taxa. At the genus level, notable genera included *Blautia*, *Escherichia–Shigella*, *Bifidobacterium*, and *Collinsella*, with differences in relative abundance across groups. To further characterize gut microbial configurations associated with cognitive status within the hypertensive population, we performed enterotype analysis restricted to the HTN-CN and HTN-aMCI groups. Enterotype analysis categorized HTN participants into two distinct microbial enterotypes (E1 and E2) (**Figure 2D**). E1 was primarily characterized by enrichment of *Blautia* and *Collinsella*, whereas E2 was dominated by *Escherichia–Shigella* (**Figure 2E**). The relative abundance of these representative genera varied notably among enterotypes, suggesting potential functional divergence in microbial community structures linked to cognitive status in hypertensive elderly individuals. Overall, these findings indicate that while community evenness remained stable, HTN-aMCI patients exhibited greater microbial richness and distinct compositional shifts, highlighting altered gut ecological structure that may contribute to cognitive impairment in the context of HTN.

### Gut microbial dysbiosis signatures discriminating HTN-aMCI patients

**Figure 3:**
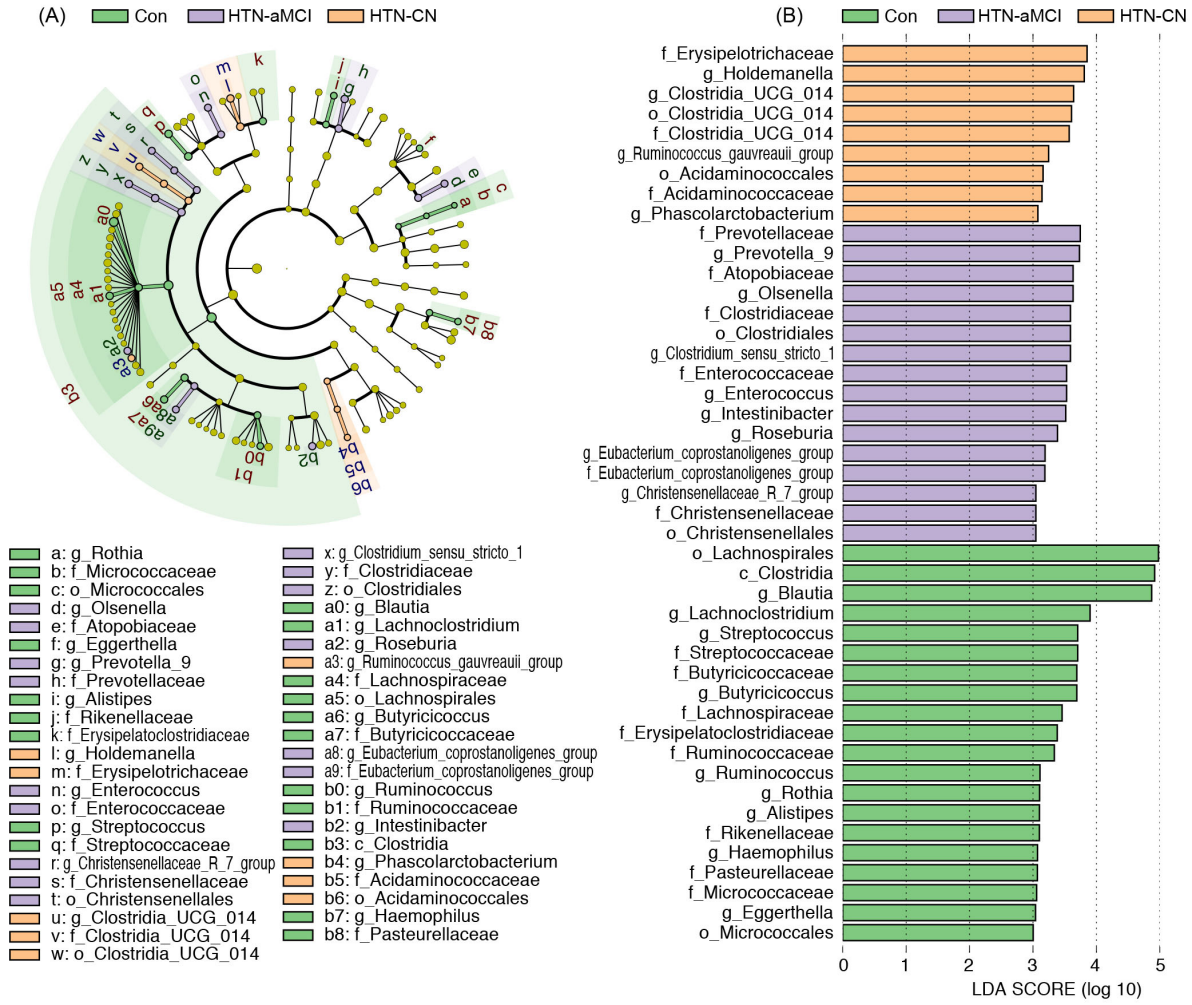
Differential gut microbial taxa associated with hypertensive patients with amnestic mild cognitive impairment (aMCI). **(A)** Cladogram generated by linear discriminant analysis effect size (LEfSe) analysis showing the phylogenetic distribution of differentially abundant bacterial taxa among the control (Con), hypertensive with normal cognition (HTN-CN), and hypertensive with aMCI (HTN-aMCI) groups. Circles radiate from the inner to outer rings representing phylum, class, order, family, and genus levels. Taxa enriched in the Con group are colored in red, those enriched in the HTN-CN group in green, and those enriched in the HTN-aMCI group in blue. Only taxa with a statistically significant difference (p < 0.05) and a linear discriminant analysis (LDA) score > 3.0 are shown. **(B)** Histogram displaying the LDA scores (log10-transformed) for the most differentially abundant bacterial taxa among the three groups (LDA score > 3.0, p < 0.05).

To identify specific microbial taxa associated with cognitive decline in hypertensive patients, we performed LEfSe analysis (LDA threshold > 3.0, p < 0.05). The cladogram illustrated the phylogenetic distribution of differentially abundant taxa across the three groups (**Figure 3A**). Compared with the Con and HTN-CN groups, the HTN-aMCI group was characterized by a significant enrichment of potentially pathogenic taxa, including *Enterococcus* (LDA = 3.53), *Clostridium*_*sensu*_*stricto*_1 (LDA = 3.59), *Christensenellaceae_R_7_group* (LDA = 3.05), and *Prevotella*_9 (LDA = 3.73) (**Figure 3B**). In contrast, SCFA-producing genera such as *Blautia*, *Butyricicoccus*, and *Ruminococcus* were significantly depleted in the HTN-aMCI group (**Figure 3B**). These results indicate that cognitive impairment in hypertensive patients is associated with a loss of beneficial commensals and an overgrowth of opportunistic pathogens.

**Figure 4:**
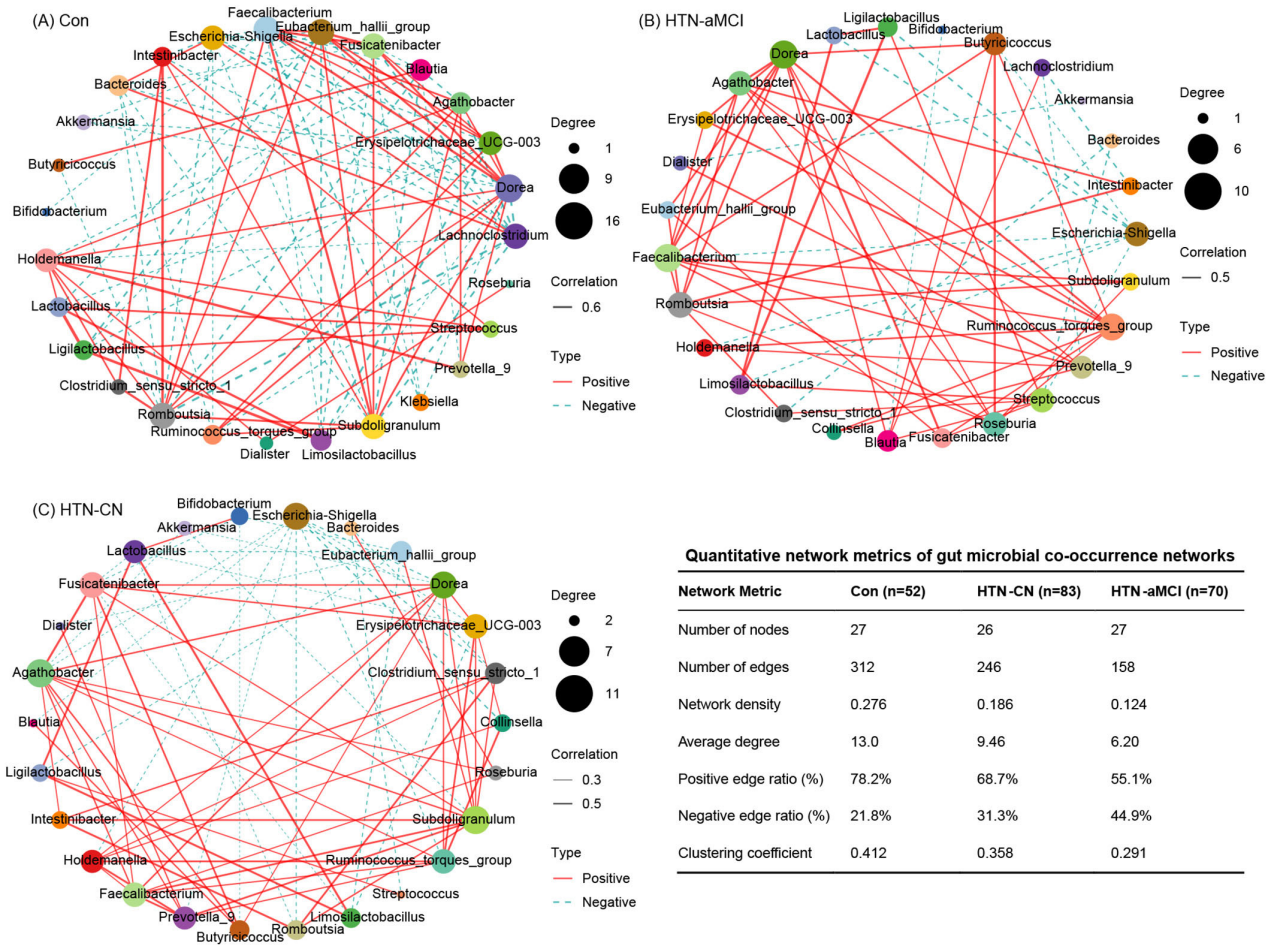
Microbial co-occurrence network analysis of gut bacterial genera across groups. Network visualization of Spearman’s correlation-based co-occurrence patterns among abundant bacterial genera (relative abundance > 0.1%) in the control (Con) group **(A)**, hypertensive with amnestic mild cognitive impairment (HTN-aMCI) group **(B)**, and hypertensive with normal cognition (HTN-CN) group **(C)**. Each node represents a bacterial genus, with node size proportional to the degree of connectivity (number of significant correlations). Red edges indicate positive correlations, and blue edges indicate negative correlations (Spearman’s correlation coefficient > 0.3 or < -0.3, p < 0.05).

To investigate whether these taxonomic shifts were accompanied by alterations in inter−bacterial ecological interactions, we constructed microbial co−occurrence networks based on Spearman’s correlations (|r| > 0.3, p < 0.05). As shown in **Figure 4**, the Con group exhibited a robust and densely connected network with 312 edges and a network density of 0.276. The HTN−CN group showed an intermediate structure (246 edges, density 0.186), whereas the HTN−aMCI group displayed a markedly sparser network with only 158 edges and a density of 0.124. Compared with the Con and HTN−CN groups, the HTN−aMCI network had significantly lower average degree (6.20 vs. 13.0 and 9.46, respectively; permutation test p < 0.01) and a marked reduction in the clustering coefficient (0.291 vs. 0.412 and 0.358). Moreover, the proportion of positive correlations decreased from 78.2% (Con) and 68.7% (HTN−CN) to only 55.1% in HTN−aMCI, while negative correlations increased correspondingly (44.9% vs. 21.8% and 31.3%). These quantitative features indicate a less cohesive, more fragmented microbial interaction network in hypertensive patients with cognitive impairment, suggesting destabilization of the gut microbial ecosystem.

**Figure 5:**
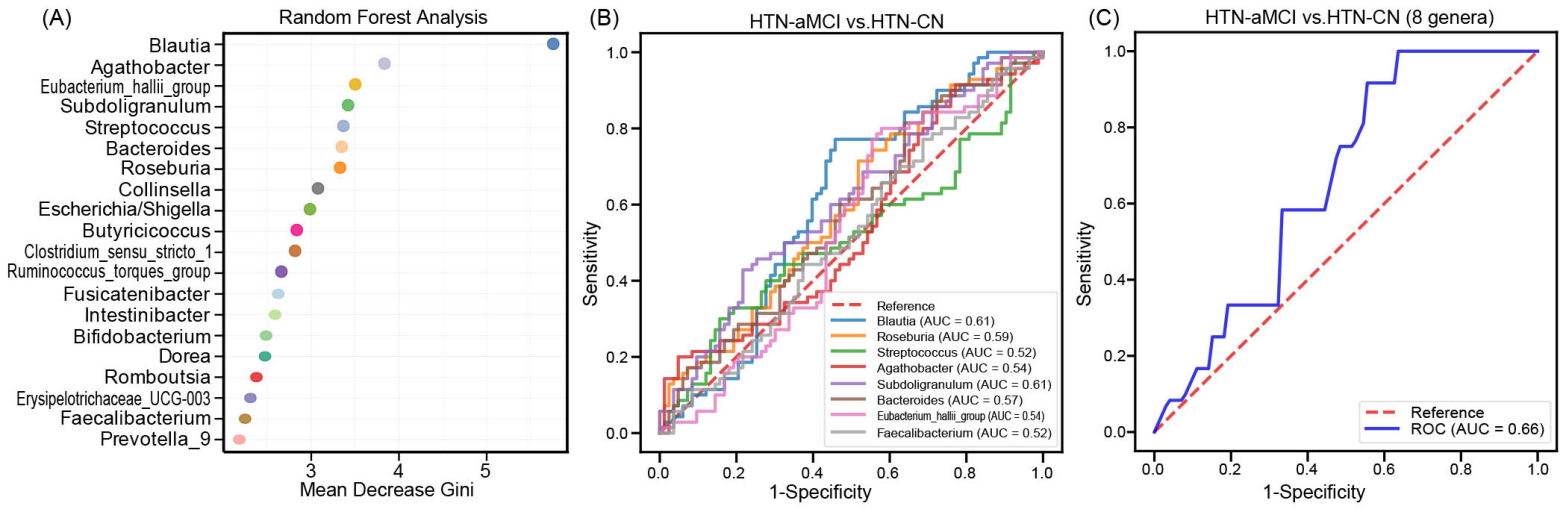
Diagnostic potential of gut microbial signatures for distinguishing hypertensive patients with amnestic mild cognitive impairment (aMCI). **(A)** Random forest analysis identifying the top bacterial genera as the most important discriminators between hypertensive patients with aMCI (HTN-aMCI) and hypertensive patients with normal cognition (HTN-CN). Variable importance was evaluated using the mean decrease Gini index. **(B)** Receiver operating characteristic (ROC) curves showing the diagnostic performance of individual microbial genera for distinguishing HTN-aMCI from HTN-CN patients. **(C)** ROC curve demonstrating the combined diagnostic performance of the 8-genus panel, which achieved an AUC of 0.66, indicating improved discriminatory ability compared with individual taxa.

To evaluate the diagnostic potential of gut microbiota for identifying aMCI in hypertensive patients, we performed random forest analysis. The model identified a panel of 8 genera as the most important discriminators between HTN-aMCI and HTN-CN groups (**Figure 5A**). Among these, depleted SCFA producers (such as *Blautia*) and enriched pathobionts (*Streptococcus*, *Escherichia-Shigella*, *Clostridium_sensu_stricto_1*) ranked as top contributors. ROC curve analysis was then conducted to assess the classification performance of individual genera and the combined panel. Among single genera, *Blautia* and *Subdoligranulum* yielded an area under the curve (AUC) of 0.61, while *Roseburia* achieved AUCs of 0.59 (**Figure 5B**). Notably, the combined panel of 8 genera demonstrated significantly improved diagnostic accuracy, with an AUC of 0.66 (**Figure 5C**). Although this multi-taxa panel outperformed individual markers, the moderate AUC values indicate that the current microbial signatures have limited standalone diagnostic power. Nevertheless, these findings suggest that gut microbial signatures, particularly the integrated panel, hold promise as non-invasive biomarkers for distinguishing HTN-aMCI from HTN-CN patients, warranting further validation in larger, independent cohorts.

Taken together, these findings demonstrate that HTN-aMCI patients harbor a distinct gut microbial dysbiosis characterized by depletion of SCFA-producing commensals, enrichment of opportunistic pathogens, and disruption of microbial co-occurrence networks, and that a multi-taxa biomarker panel can effectively discriminate HTN-aMCI from HTN-CN patients.

### Functional alterations in gut microbiota associated with HTN-aMCI

To investigate whether the observed taxonomic shifts were accompanied by alterations in microbial functional potential, we performed PICRUSt2-based functional prediction and compared KEGG pathway abundances across the three groups using pairwise comparisons.

**Figure 6:**
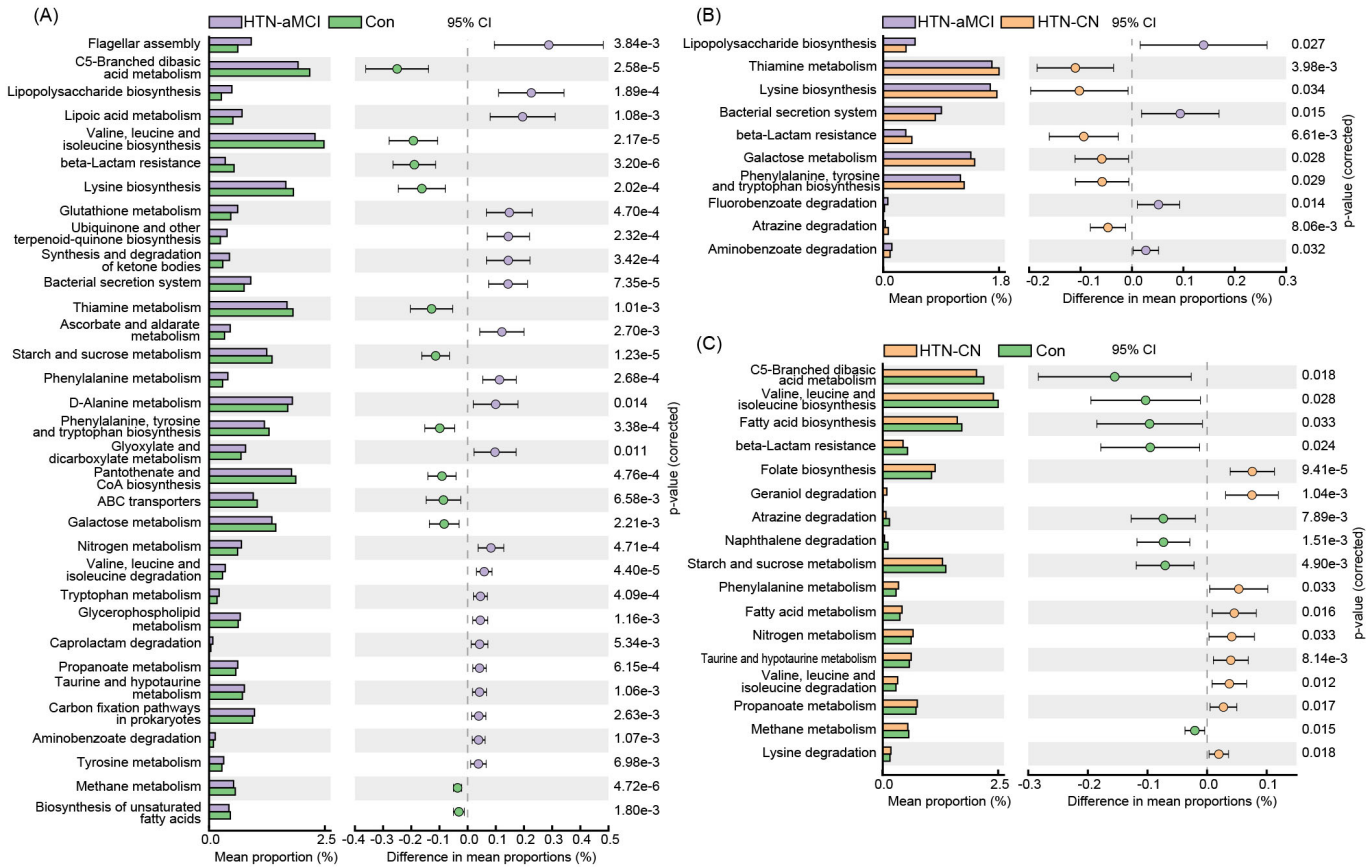
Predicted functional alterations of gut microbiota in hypertensive patients with aMCI. PICRUSt2 analysis comparing KEGG pathways among the Con, HTN-CN, and HTN-aMCI groups (Welch’s t-test, FDR-corrected). **(A)** HTN-aMCI vs. Con: enrichment of pro-inflammatory pathways (methane metabolism, tyrosine degradation, LPS biosynthesis, bacterial secretion systems, beta-lactam resistance) and depletion of carbohydrate metabolism pathways (starch/sucrose metabolism, galactose metabolism). **(B)** HTN-aMCI vs. HTN-CN: enrichment of xenobiotic degradation pathways, LPS biosynthesis, bacterial secretion, and beta-lactam resistance. **(C)** HTN-CN vs. Con: enrichment of methane metabolism, propanoate metabolism, and amino acid degradation pathways. A progressive functional shift from Con to HTN-CN to HTN-aMCI was observed.

In the comparison between HTN-aMCI and Con groups (**Figure 6A**), the HTN-aMCI group showed significant enrichment of pathways linked to inflammation and microbial pathogenicity. Lipoic acid metabolism, Phenylalanine metabolism, D-Alanine metabolism, Glycerophospholipid metabolism, Propanoate metabolism, and Caprolactam degradation were all elevated. Notably, LPS biosynthesis, and bacterial secretion systems were also enriched, indicating enhanced pro-inflammatory potential and increased pathogenic activity. Conversely, pathways related to carbohydrate metabolism, including starch and sucrose metabolism and galactose metabolism, were reduced, suggesting diminished SCFA-producing capacity.

In the comparison between HTN-aMCI and HTN-CN groups (**Figure 6B**), the HTN-aMCI group exhibited enrichment of pathways directly associated with inflammation and bacterial virulence. Aminobenzoate degradation, and fluorobenzoate degradation—pathways involved in xenobiotic metabolism and oxidative stress responses—were elevated. Beta-lactam resistance, bacterial secretion systems, and LPS biosynthesis were also significantly increased, further supporting a pro-inflammatory shift in the gut microbiota of HTN-aMCI patients. Additionally, lysine biosynthesis and thiamine metabolism were enriched, while galactose metabolism was reduced.

In the comparison between HTN-CN and Con groups (**Figure 6C**), the HTN-CN group showed enrichment of lysine degradation, propanoate metabolism, taurine and hypotaurine metabolism, nitrogen metabolism, fatty acid metabolism, and phenylalanine metabolism. Pathways related to starch and sucrose metabolism, valine, leucine and isoleucine biosynthesis and fatty acid biosynthesis were reduced. Notably, several pathways enriched in HTN-aMCI compared with Con, such as phenylalanine metabolism, were also elevated in HTN-CN relative to Con, suggesting that HTN itself drives a partial functional shift that is further amplified with cognitive decline.

Thus, these findings demonstrate that HTN-aMCI patients harbor a gut microbiota with enhanced potential for LPS biosynthesis, bacterial secretion, and xenobiotic metabolism, all of which are closely linked to systemic inflammation. The progressive enrichment of these pro-inflammatory pathways from Con to HTN-CN to HTN-aMCI supports a stepwise functional deterioration associated with HTN and subsequent cognitive impairment.

### Associations between gut microbiota and systemic inflammation

Systemic inflammatory profiles were assessed by measuring 27 cytokines, chemokines, and growth factors in serum using a multiplex immunoassay. Compared with the HTN-CN group, the HTN-aMCI group exhibited significantly elevated levels of multiple pro-inflammatory mediators. These were grouped into functional axes: Th1-related cytokines (IL-2; TNF-α), Th2-related cytokines (IL-4; IL-5), and most notably Th17-axis mediators (IL-17; IL-6; IL-1β). Chemokines, including IP-10, RANTES, and MIP-1α, as well as growth factors G-CSF, GM-CSF, and PDGF-bb, were also significantly higher in the HTN-aMCI group. These results indicate that cognitive decline in hypertensive individuals is associated with an exacerbated systemic inflammatory state, with a prominent Th17 signature.

**Figure 7:**
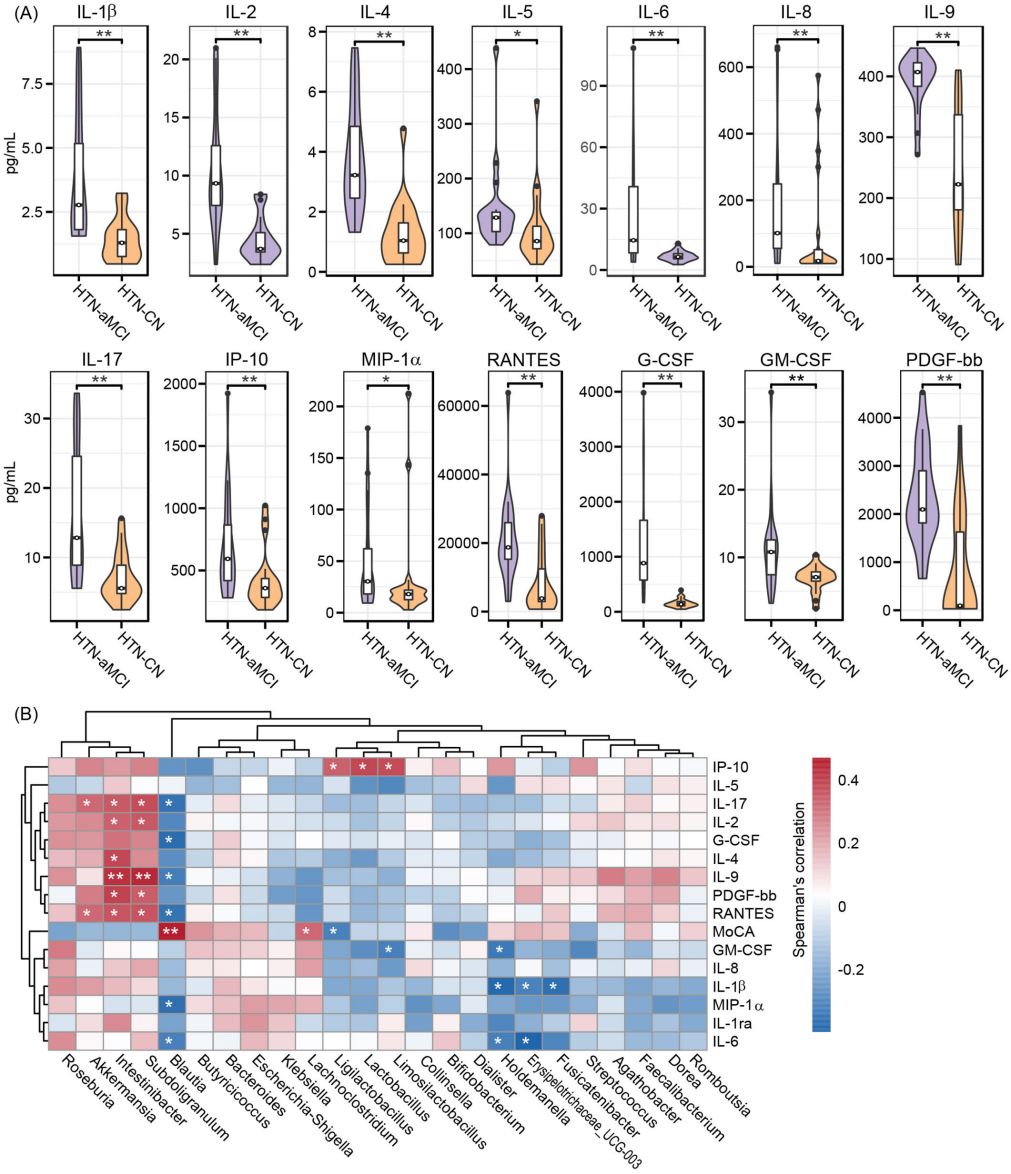
Systemic inflammation and its correlation with gut microbiota in hypertensive patients with aMCI. **(A)** Serum levels of pro-inflammatory cytokines and chemokines in HTN-CN and HTN-aMCI groups. The HTN-aMCI group showed significantly elevated levels of IL-1β, IL-2, IL-4, IL-5, IL-6, IL-8, IL-9, IL-17, IP-10, MIP-1α, RANTES, G-CSF, GM-CSF, and PDGF-bb compared with the HTN-CN group (*p < 0.05, **p < 0.01). **(B)** Spearman’s correlation heatmap between differentially abundant microbial genera and inflammatory markers or MoCA scores. *Blautia* showed negative correlations with pro-inflammatory cytokines (IL-6, IL-8, IL-9, IL-17, MIP-1α, RANTES) and a positive correlation with MoCA scores. In contrast, *Intestinibacter* and *Subdoligranulum* exhibited positive correlations with IL-17 and RANTES (*p < 0.05, **p < 0.01).

To explore the relationship between gut microbiota and systemic inflammation, we performed Spearman’s rank correlation analysis was performed to assess the associations between differentially abundant microbial genera, measured inflammatory markers, as well as MoCA scores (**Figure 7B**). Among SCFA-producing genera, *Blautia* showed significant negative correlations with IL-6, IL-8, IL-9, IL-17, MIP-1α, and RANTES, as well as a positive correlation with MoCA scores, suggesting a potential protective role in mitigating inflammation and cognitive impairment. Similarly, *Lachnoclostridium* exhibited a positive correlation with MoCA scores, further supporting the beneficial role of SCFA-producing taxa in cognitive function. *Erysipelotrichaceae_UCG-003* and *Holdemanella* were negatively correlated with IL-1β and IL-6, while *Fusicatenibacter* showed a negative correlation with IL-1β, indicating their potential involvement in regulating pro-inflammatory responses. In contrast, several potentially pathogenic genera displayed opposing correlation patterns: *Akkermansia*, *Intestinibacter*, and *Subdoligranulum* showed significant positive correlations with IL-17 and RANTES, with *Intestinibacter* and *Subdoligranulum* additionally demonstrating positive correlations with IL-2 and IL-9. Furthermore, *Ligilactobacillus*, *Lactobacillus*, and *Limosilactobacillus* exhibited positive correlations with IP-10, a key chemokine involved in inflammatory cell recruitment. No significant correlations with the measured cytokines or MoCA scores were observed for the remaining genera, including *Roseburia*, *Butyricicoccus*, *Bacteroides*, *Escherichia-Shigella*, *Klebsiella*, *Collinsella*, *Bifidobacterium*, *Dialister*, *Streptococcus*, *Agathobacter*, *Faecalibacterium*, *Dorea*, and *Romboutsia*.

Overall, these correlation patterns suggest that the depletion of SCFA-producing commensals (e.g., *Blautia*) and enrichment of opportunistic pathogens (e.g., *Intestinibacter*, *Subdoligranulum*) are specifically associated with systemic inflammation and cognitive decline in hypertensive patients. These findings provide a potential mechanistic link between gut microbial dysbiosis and the pro-inflammatory state observed in HTN-aMCI, highlighting the role of the gut-brain axis in the pathogenesis of cognitive impairment in hypertension.

## Discussion

Our present study provides a comprehensive characterization of gut microbial dysbiosis in elderly Chinese HTN-aMCI patients, revealing an interconnected triad of pathologies: disruption of the gut ecosystem, systemic inflammation, and cognitive decline. Our findings indicate that HTN-aMCI patients possess a distinctive gut microbiota profile characterized by a reduction in SCFA-producing commensals, such as *Blautia*, *Butyricicoccus*, and *Faecalibacterium*, alongside an enrichment of opportunistic pathogens, including *Clostridium sensu stricto* 1, and *Enterococcus*. Furthermore, the microbial co-occurrence network in these patients appears fragmented. These microbial alterations correlate with elevated levels of pro−inflammatory cytokines and chemokines, suggesting that gut dysbiosis may be associated with cognitive impairment by exacerbating systemic inflammation.

The intersection of hypertension and cognitive impairment poses a significant public health challenge, particularly among aging populations. Hypertension is widely recognized as a modifiable risk factor for dementia, with mechanisms that include cerebrovascular damage, neurovascular uncoupling, and chronic inflammation (40, 41). However, the considerable variability in cognitive outcomes among hypertensive individuals implies the presence of additional modifying factors. In this context, our study positions gut microbiota as a possible biological mediator, aligning with the emerging paradigm of the gut-brain axis (42).

The notable depletion of SCFA-producing genera in our HTN-aMCI cohort is particularly significant. SCFAs, primarily acetate, propionate, and butyrate, are critical metabolites that uphold intestinal barrier integrity, modulate immune responses, and exert neuroprotective effects (43–46). Butyrate, specifically, has been shown to enhance tight junction integrity between intestinal epithelial cells, thus preventing the translocation of bacterial endotoxins into systemic circulation (47). Additionally, SCFAs can cross the blood-brain barrier, influencing microglial maturation and function (48). The substantial reduction of *Blautia*, and *Butyricicoccus* in our HTN-aMCI patients is consistent with earlier findings in both hypertension and cognitive impairment (6, 49, 50). Verhaar et al. reported that hypertensive individuals consistently exhibit lower abundances of SCFA-producing microbiota and higher levels of Gram-negative bacteria, which serve as a source of LPS (49). When we examine the three groups together, a clear gradient of dysbiosis emerges. The loss of SCFA producers is mild in HTN-CN but becomes markedly more severe in HTN-aMCI, while pro-inflammatory taxa and pathways show the opposite trend. This stepwise deterioration suggests that the gut microbiota does not shift abruptly at the onset of cognitive impairment but rather degrades progressively along a continuum of vascular and neurodegenerative burden. Our findings thus extend previous work by demonstrating that the added burden of cognitive impairment correlates with an even more pronounced loss of these beneficial taxa, reinforcing the concept of a disease-severity-linked gradient. Importantly, recent studies have consistently observed similar patterns of SCFA-producer depletion across the cognitive decline spectrum. Donaldson et al. demonstrated that individuals with AD had significantly decreased proportions of *Faecalibacterium*, *Blautia*, and *Roseburia* species compared to controls without dementia (51). This parallel is striking, as the same three genera were depleted in our HTN-aMCI patients, suggesting a shared microbial signature of cognitive impairment regardless of the underlying primary pathology (hypertension versus idiopathic AD). Furthermore, a large Korean study involving 346 older individuals reported that *Bifidobacterium catenulatum*, *Anaerobutyricum hallii*, and *Anaerostipes hadrus* were elevated in subjective cognitive decline, with progressive loss of community complexity as cognitive status worsened from mild cognitive impairment to AD (52). These findings collectively indicate that depletion of butyrate-producing taxa may represent a convergent microbial phenotype across diverse etiologies of cognitive decline.

The concurrent enrichment of potentially pathogenic taxa, such as *Clostridium sensu stricto* 1, and *Enterococcus*, in HTN-aMCI patients raises the possibility that these organisms may be associated with neuroinflammation. These genera are known to contain LPS or other pathogen-associated molecular patterns (PAMPs) that activate Toll-like receptors (TLRs) on intestinal epithelial cells and immune cells, triggering the release of pro-inflammatory cytokines (53). Donaldson et al. similarly reported significantly increased proportions of *Clostridium_sensu_stricto*_1 in AD patients compared to controls, alongside elevated fecal calprotectin, a biomarker of gut inflammation (51). Qu et al. reported that hypertensive patients with cognitive impairment exhibited significant increases in LPS-containing taxa, including Proteobacteria and *Escherichia-Shigella*, which correlated with downregulation of glycan biosynthesis and metabolic pathways (50). This pro-inflammatory shift has been observed in cognitive impairment, as a review by Hawkins et al. concluded that depletion of SCFA-producing taxa (*Faecalibacterium*, *Roseburia*, *Eubacterium*) and elevations in inflammatory mediators (IL-6, TNF-α, IL-1β, IL-17) are shared across neuropsychiatric disorders and their comorbidities (54). Our study similarly identified functional enrichment of LPS biosynthesis and bacterial secretion systems in HTN-aMCI patients, establishing a mechanistic link between taxonomic shifts and inflammatory potential.

One of the most novel findings of our study is the disrupted microbial co-occurrence network identified in HTN-aMCI patients, which aligns with recent ecological studies of the gut microbiota in neurodegeneration. Network analysis transcends simple comparisons of individual taxon abundances, focusing instead on the ecological stability of the gut microbial community. The fragmented network observed in HTN-aMCI patients, marked by diminished positive interactions among commensals and heightened negative correlations involving pathobionts, indicates a destabilized ecosystem that is less resilient to perturbations. To our knowledge, this is the first report of such ecological destabilization specifically in elderly HTN-aMCI patients. Xin et al. recently demonstrated a stepwise decline in network complexity across cognitive states, with mild cognitive impairment exhibiting the highest vulnerability and lowest robustness, representing a critical transitional state (55). Notably, their study showed that the MCI network displayed a complete absence of keystone taxa, whereas the AD network retained only connector species, indicating a fundamental reorganization of the gut microbial ecosystem during disease progression. Son et al. similarly reported greater microbial community complexity in subjective cognitive decline compared to MCI and AD groups (52). Importantly, this network-level disruption was absent in HTN-CN group, suggesting that the added burden of aMCI drives a distinct ecological collapse beyond the changes induced by hypertension alone. This ecological collapse could be part of a cycle in which dysbiosis is associated with increased gut permeability, systemic inflammation with impaired cerebral vascular function, and cognitive decline with altered dietary habits and stress responses, thereby exacerbating dysbiosis (56, 57). Similar network-level disruptions have been reported in other neurological disorders, including AD and PD (57, 58), and our findings extend this concept to cognitive impairment associated with hypertension, thereby establishing disrupted microbial co-occurrence networks as a key ecological signature and a potential novel mechanistic link in the gut-brain axis.

The systemic inflammatory profile observed in our HTN-aMCI group, characterized by elevated levels of IL-1β, IL-6, IL-8, IL-17, IP-10, and MIP-1α, aligns with the concept of “inflammaging”, a chronic low-grade inflammatory state that accelerates cognitive decline (12, 59). The most pronounced elevation in HTN−aMCI patients was observed in the Th17 axis (IL−17, IL−6, IL−1β), together with significant increases in Th1−related (IL−2, TNF−α) and Th2−related (IL−4, IL−5) cytokines. This Th17-skewed profile is pathophysiologically relevant, as IL-17 can compromise blood-brain barrier integrity, promote neuroinflammation, and synergize with IL-6 and IL-1β to sustain central nervous system immune activation (53, 60). Importantly, recent evidence has established that individuals with mild cognitive impairment exhibit a distinct “inflammatory fingerprint” characterized by elevated IL-17A, IL-6, and TNF-α, which can be reversed by targeted interventions, underscoring the dynamic nature of this peripheral immune dysregulation (61). In our study, the Th17-dominant pattern was not an isolated finding; rather, it correlated strongly with gut microbial shifts. As shown in our correlation analyses (**Figure 7B**), the depletion of SCFA-producing *Blautia* was negatively associated with Th17-related cytokines (IL-17, IL-6), whereas pathobionts such as *Intestinibacter* and *Subdoligranulum* showed positive correlations with IL-17 and RANTES, suggesting that gut dysbiosis may drive a Th17-skewed systemic response (62–64). This grouped interpretation thus provides a clearer mechanistic link between microbiota alterations and cognitive decline, reinforcing the potential of targeting Th17-driven inflammation as a therapeutic avenue in HTN-aMCI (65).

Emerging mechanistic insights have significantly advanced our understanding of how gut microbiota and their metabolites drive neuroinflammation. A landmark study by Blackmer-Raynolds et al. demonstrated that intestinal colonization with *Escherichia coli* alone is sufficient to induce a distinct myeloid cell activation state, increase brain-resident CD8^+^ T cells, and exacerbate cognitive decline and amyloid pathology in 5×FAD mice, providing definitive causal evidence that specific gut microbes can directly modulate neurodegenerative disease susceptibility (66). Furthermore, a systematic review by Borrego-Ruiz and Borrego has highlighted that microbial metabolites, particularly SCFAs and tryptophan derivatives, are key regulators of neuroinflammation, lipid metabolism, and gene expression, with their dysregulation directly impacting AD pathology (67). Verosky et al. further elucidated that microglia, the brain’s resident immune cells, are exquisitely sensitive to microbial metabolites; SCFAs, indoles, and secondary bile acids program microglial development and function, whereas pro-inflammatory metabolites like trimethylamine N-oxide disrupt microglial homeostasis and worsen neurodegeneration (68). These findings mechanistically support our observation that depletion of SCFA-producing *Blautia* and *Butyricicoccus* correlates with heightened systemic inflammation, likely through loss of tonic microbial suppression of microglial activation. Additionally, Solorzano et al. recently reported in a memory clinic population that lower cognitive functioning specifically associates with higher abundance of pro-inflammatory taxa (*Dialister*, *Clostridia*_UCG-014) and elevated LPS levels, providing clinical validation of the gut–inflammation–cognition axis we observe in HTN-aMCI patients (69). Together, these recent studies corroborate our findings and position gut microbiota as a factor that is correlated with neuroinflammation, which in turn is associated with cognitive decline in hypertensive individuals.

From a diagnostic standpoint, our random forest model identified an eight-genus panel that distinguished HTN-aMCI from HTN-CN with an area under the curve (AUC) of 0.66. While this level of diagnostic accuracy is moderate and not yet clinically actionable, it is comparable to or exceeds many single-biomarker approaches for complex multifactorial conditions. The potential of gut microbiome signatures for diagnosing cognitive impairment in hypertension has been previously suggested. For instance, Qu et al. achieved an excellent AUC of 0.944 using a nine-taxa panel; however, the smaller sample size and potential for overfitting in that study necessitate cautious interpretation (50). Our larger cohort (n=205) provides a more robust estimate of diagnostic performance in real-world scenarios. Future research integrating metagenomic sequencing for enhanced taxonomic and functional resolution, combined with targeted metabolomics for SCFAs and other microbial metabolites, may significantly improve diagnostic accuracy.

Functional predictions derived from our PICRUSt2 analysis revealed an enrichment of pro-inflammatory pathways (including methane metabolism, tyrosine degradation, and LPS biosynthesis) and a depletion of SCFA biosynthesis pathways in HTN-aMCI patients. Notably, some of these functional shifts were already present in HTN-CN patients but were amplified in HTN-aMCI, suggesting a progressive functional deterioration. This observation aligns with the notion that hypertension primes the gut-brain axis for dysfunction, with additional factors, whether genetic, environmental, or stochastic, precipitating cognitive decline (70). Huang et al. similarly identified altered benzoate degradation and phenylpropanoid biosynthesis pathways in hypertensive patients experiencing cognitive decline using metabolomics (41), which corroborates our functional predictions and highlights the consistency of metabolic pathway alterations across independent cohorts.

Several limitations of this study must be acknowledged. First, the cross-sectional design limits causal inference. While we hypothesize that dysbiosis contributes to cognitive impairment, the observed associations could also indicate reverse causation (where cognitive decline alters diet or stress, subsequently shaping the microbiota) or could be confounded by unmeasured variables. Longitudinal studies with repeated microbiota sampling are essential to establish temporal relationships. Second, our functional predictions using PICRUSt2, while informative, are based on 16S rRNA data and lack the resolution of shotgun metagenomic sequencing. Direct measurements of fecal SCFAs, LPS, and other metabolites would bolster mechanistic conclusions. Third, medication use, particularly antihypertensive agents (ACE inhibitors/angiotensin receptor blockers, utilized by approximately 63% of HTN patients), antibiotics and probiotics, may influence gut microbiota composition. Although we did not observe significant differences in medication use between the HTN-CN and HTN-aMCI groups, subtle medication effects cannot be completely ruled out. Fourth, our study population consisted exclusively of elderly Chinese individuals from a single geographic region (Lishui, Zhejiang), which may limit the generalizability of our findings to other ethnicities and settings, given the well-established influence of diet and geography on gut microbiota. Lastly, while we excluded individuals with major neurological or gastrointestinal diseases, residual confounding from subclinical conditions or lifestyle factors (such as dietary intake, physical activity, and sleep) cannot be entirely excluded (71).

The therapeutic implications of our findings are significant. The depletion of SCFA-producing bacteria in HTN-aMCI patients suggests that dietary interventions designed to increase the intake of fermentable fibers (such as resistant starch, inulin, and pectins) could restore beneficial microbial populations and enhance SCFA production. Probiotic supplementation with strains of *Roseburia*, *Blautia*, or *Faecalibacterium*, though not yet commercially available—represents a targeted approach that warrants investigation. Preclinical studies have demonstrated that acetate supplementation can prevent hypertension and heart failure in hypertensive mice, and that *Clostridium butyricum* can improve cognitive function in vascular dementia models through SCFA-mediated mechanisms (50, 72). Fecal microbiota transplantation (FMT) has shown promise in modulating neuroinflammation in animal models of AD, with pilot human studies currently underway (73, 74). However, the translation of these findings to clinical practice will necessitate rigorous safety and efficacy trials.

In conclusion, this study underscores that elderly hypertensive patients with aMCI exhibit a distinct gut microbial dysbiosis characterized by the loss of SCFA-producing commensals, an expansion of pathobionts, disrupted microbial networks, and enhanced pro-inflammatory functional potential. These microbial alterations are strongly associated with systemic inflammation and cognitive performance, reinforcing the significance of the gut-brain axis in hypertension-related cognitive impairment. Our findings lay the groundwork for future longitudinal and interventional studies aimed at establishing causality and exploring microbiota-targeted therapies as promising strategies to preserve cognitive function in the increasingly prevalent population of elderly hypertensive individuals.

## Data Availability

The datasets presented in this study can be found in online repositories. The names of the repository/repositories and accession number(s) can be found in the article/supplementary material.
